# Assessing the suitability of capillary electrophoresis‐mass spectrometry for biomarker discovery in plasma‐based metabolomics

**DOI:** 10.1002/elps.201900126

**Published:** 2019-05-02

**Authors:** Wei Zhang, Karen Segers, Debby Mangelings, Ann Van Eeckhaut, Thomas Hankemeier, Yvan Vander Heyden, Rawi Ramautar

**Affiliations:** ^1^ Biomedical Microscale Analytics Division of Systems Biomedicine and Pharmacology Leiden Academic Centre for Drug Research Leiden University The Netherlands; ^2^ Department of Analytical Chemistry Applied Chemometrics and Molecular Modelling Vrije Universiteit Brussel Brussel Belgium; ^3^ Department of Pharmaceutical Chemistry Drug Analysis and Drug Information Center for Neurosciences Vrije Universiteit Brussel Brussel Belgium

**Keywords:** Mass spectrometry, Metabolic profiling, Metabolomics, Spiked biomarkers, Validation

## Abstract

The actual utility of capillary electrophoresis‐mass spectrometry (CE‐MS) for biomarker discovery using metabolomics still needs to be assessed. Therefore, a simulated comparative metabolic profiling study for biomarker discovery by CE‐MS was performed, using pooled human plasma samples with spiked biomarkers. Two studies have been carried out in this work. Focus of study I was on comparing two sets of plasma samples, in which one set (class I) was spiked with five isotope‐labeled compounds, whereas another set (class II) was spiked with six different isotope‐labeled compounds. In study II, focus was also on comparing two sets of plasma samples, however, the isotope‐labeled compounds were spiked to both class I and class II samples but with concentrations which differ by a factor two between both classes (with one compound absent in each class). The aim was to determine whether CEMS‐based metabolomics could reveal the spiked biomarkers as the main classifiers, applying two different data analysis software tools (MetaboAnalyst and Matlab). Unsupervised analysis of the recorded metabolic profiles revealed a clear distinction between class I and class II plasma samples in both studies. This classification was mainly attributed to the spiked isotope‐labeled compounds, thereby emphasizing the utility of CE‐MS for biomarker discovery.

**Abbreviations**
FDR: False discovery rate
IS: Internal standard
LOOCV: Leave‐one‐out cross‐validation
MCR‐ALS: Multivariate curve resolution ‐ alternating least squares
MSI: Multi‐segment injection
PCA: Principal component analysis
PLS‐DA: Partial least squares ‐ discriminant analysis
QC: Quality control
ROI: Region of interest
VIP: Variable importance in projection

## 1 Introduction

**Figure 1:**
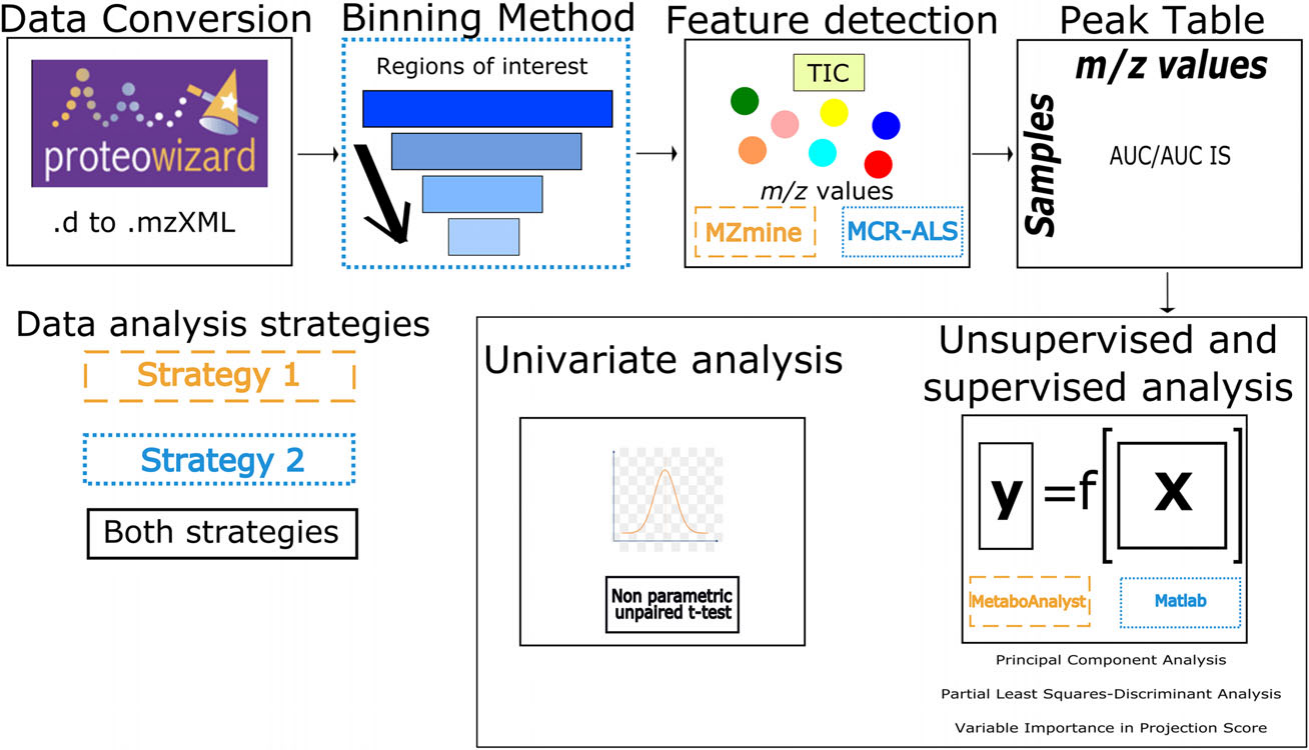
Overview of the data analysis tools used in this study. The tools of the first data analysis strategy are shown in orange (stripes) while in blue (dots) those for the second strategy are given. The workflow is similar starting with the data conversions to a readable file. Subsequently, data compression is needed for the MCR‐ALS feature detection of the second strategy. After selecting the features a peak table is generated containing the corrected peak areas for each sample. The generated peak table can be further investigated using univariate, unsupervised and supervised analysis to discover potential biomarkers.

Metabolomics offers a new approach to explore changes in patterns for a large number of (endogenous) metabolites in biological media, such as blood, urine, and cerebrospinal fluid.^1^, ^2^, ^3^, ^4^, ^5^, ^6^ Currently, a wide range of advanced analytical separation techniques is used for metabolic profiling of biological samples. The complex data sets generated by these analytical tools can be processed by software tools, for example XCMS,^7^ MZmine,^8^ MetAlign,^9^ or SpectConnect,^10^ and the main output is a peak table with the intensity of each chromatographic or electrophoretic peak, characterized by a specific retention or migration time, respectively, and one or more *m/z* values. Supervised and unsupervised chemometric approaches are often used to get visualization of the relations between the metabolic profiles and to define borders between groups of samples. Global profiling of (endogenous) metabolites in organisms has been vastly explored for its potential application in research areas, such as diagnosis of diseases,^1^, ^3^, ^6^ guidance for personalized medicine,^11^ and evaluation of therapeutic treatments.^12^, ^13^ Despite the efforts dedicated to metabolomics for biomarker discovery, its impact on recent clinical practice is still rather limited due to various challenges encountered during the analytical process, including study design, sample handling, data acquisition and data analysis,^14^ which may potentially lead to contradictory results in reported biomarkers. For example, Slupsky et al.^15^ indicated succinic acid to be among the down‐regulated urinary metabolites in ovarian cancer patients, whereas Zhang et al.^16^ obtained the opposite finding for this compound using a different analytical technique. Therefore, these studies clearly underscore the need for assessing the capability of a given analytical technique for delivering the right biomarkers in metabolomics using preferably multiple data analysis procedures. In principle, each data analysis procedure should provide the same chemical information/output when employing a single analytical technique for metabolic profiling. In this work, we have used MetaboAnalyst and Matlab as two data analysis software tools for analyzing metabolomics data obtained by CE–MS (Fig. 1).

CE is a separation technique that is well‐suited for the highly efficient profiling of polar and charged metabolites, as compounds are separated according to their charge‐to‐size ratios. It provides complementary metabolic information compared to chromatography‐based techniques. Until now, CE coupled to MS has been utilized for metabolic profiling of a wide range of biological samples in various application fields.^17^ However, in comparison to other analytical techniques the use of CE–MS in metabolomics is still underrepresented.^18^ CE–MS is often still considered by the scientific community as a rather complicated or not robust technique, in this case specifically the coupling of CE to MS, and often not fulfilling the criteria of repeatability and sensitivity for metabolomics studies.

Over the past years, various studies have clearly indicated the long‐term performance of CE–MS for metabolomics and peptide profiling studies.^19^, ^20^, ^21^ For example, the group of Soga and co‐workers, who introduced the first CE–MS methods for metabolomics in 2003,^22^ has recently assessed the long‐term performance of CE–MS for metabolic profiling of more than 8000 human plasma samples from the Tsuruoka Metabolomics Cohort Study over a 52‐month period.^20^ The study provided an absolute quantification of 94 polar metabolites in plasma with a similar or better reproducibility than other analytical platforms employed for large‐scale metabolomics studies. The issue of migration‐time repeatability for metabolic profiling studies can be tackled by converting migration times into electrophoretic mobilities, as recently demonstrated by Drouin et al. 23. This group has designed an experimental database for effective mobilities (μ_eff_) measured for a large variety of charged metabolites, which was successfully implemented for the characterization of metabolites observed by CE–MS in tumor cell samples. Overall, we expect that this approach will be of great value for metabolomics studies, especially for the identification of metabolites when employing a library based on electrophoretic mobilities. Other improvements in CE–MS analyses, such as the use of novel interfaces^24^, ^25^ and multi‐segment injection (MSI),^26^ have clearly contributed to the potential of CE–MS of becoming a sensitive and high‐throughput technique for metabolic profiling studies. Apart from increasing sample throughput, the MSI approach, developed by the group of Britz‐McKibbin,^26^ could also be used to distinguish authentic metabolite features from spurious signals in biological samples. The latter could readily be annotated based on their temporal signal pattern when using the MSI approach in combination with high‐resolution tandem mass spectrometry.

Up till now, CE–MS has been used by various research groups for a wide range of metabolomics studies providing useful insights into questions/problems from different fields. Still, it is important to show the actual utility of CE–MS for comparative metabolic profiling studies, especially in order to convince the scientific community about the usefulness of this approach for biomarker discovery. An artificial metabolomics study was therefore designed to test the capability of CE–MS in finding the correct biomarkers in a comparative metabolic profiling study. For this, two studies have been carried out, in which the focus of study I was on comparing two sets of plasma samples, i.e., class I was spiked with five isotope‐labeled compounds, whereas class II was spiked with six different isotope‐labeled compounds. In study II, the focus was also on comparing two sets of plasma samples, however, in this case the isotope‐labeled compounds were spiked to both class I and class II samples but with concentrations which differ by a factor two between both classes, and with the absence of one compound in each class. Blank pooled human plasma (without spiking) was used as quality control (QC) sample to assess the performance of CE–MS over time. Overall, the strategy outlined in this paper could be considered as an approach to validate a (conventional) CE–MS method for metabolomics studies.

## 2 Materials and methods

### 2.1 Chemicals and reagents

HPLC grade methanol and acetonitrile were obtained from Actu‐All Chemicals (Oss, the Netherlands). HPLC grade chloroform was provided by Biosolve Chemicals (Valkensweerd, the Netherlands). Acetic acid (99–100%) and sodium hydroxide were purchased from VWR (Amsterdam, the Netherlands). Ammonium hydroxide (28–30%) was acquired from Acros Organics (Amsterdam, the Netherlands). Water in this work was produced by a Milli‐Q® Advantage A10 Water Purification System from Millipore (Amsterdam‐Zuidoost, the Netherlands). The standards of eleven ^13^C, ^15^N and/or D‐isotope‐labeled amino acids were purchased from Cambridge Isotope Laboratories (Apeldoorn, the Netherlands). In Study I, DL‐phenyl‐D5‐alanine from CDN ISOTOPES (Nieuwegein, the Netherlands) was used as the internal standard (IS). In study II, an L‐methionine sulfone‐containing solution from Human Metabolome Technologies (Leiden, the Netherlands) was employed as IS. All compounds were dissolved in a mixture of water:acetonitrile (95:5, containing 0.5% v/v formic acid) and subsequently diluted to desired concentrations with water (see Tables 1 and 2). A solution of acetic acid (10% v/v in water, pH = 2.2) was employed as BGE.

**Table 1:** An overview of the design of class I and class II plasma samples for study I (IS: DL‐phenyl‐D5‐alanine). Sample 1 within class I is prepared by spiking Mix 1 to the blank plasma sample, and sample 2 within class I is prepared by spiking Mix 2 to the plasma sample, etc

			Concentration (µM)				
		Class I (*n* = 6 samples per mix)						
Compound	*m/z*	Mix 1	Mix 2	Mix 3		Mix 4	Mix 5	
L‐Isoleucine (13C; 15N)	134.099	40	36	50		40	36	
L‐Asparagine (13C2;15N2)	139.066	100	80	90		80	80	
L‐Glutamine (13C2)	149.081	20	15	30		30	30	
L‐Lysine (4,4,5,5‐D4)	151.135	10	10	15		12	15	
L‐Tryptophan (13C11;15N2)	218.124	40	48	50		36	50	
		Class II (*n* = 5 samples per mix)						
		Mix 6	Mix 7	Mix 8	Mix 9	Mix 10	Mix 11	
Creatinine (N‐methyl‐D3)	117.088	40	30	45	50	45	50	
L‐Valine (D5)	126.134	5	7.5	10	7.5	10	7.5	
L‐Asparagine (2,3,3‐D3)	136.078	100	80	90	100	80	90	
L‐Glutamine (2,3,3,4,4‐D5)	152.110	100	90	100	80	90	80	
L‐Lysine (13C6)	153.129	40	35	50	45	35	40	
L‐Glutamic acid (13C5;D5;15N)	159.103	40	45	30	50	30	40	

**Table 2:** Design of class I and class II plasma samples for study II (IS: L‐methionine sulfone)

		Class I (*n* = 30 samples)	Class II (*n* = 30 samples)
Compound	*m/z*	Concentration (µM)	Concentration (µM)
L‐Lysine (4,4,5,5‐D4)	151.135	20	10
L‐Asparagine (13C2;15N2)	139.066	100	50
L‐Isoleucine (13C; 15N)	134.099	20	40
L‐Tryptophan (13C11;15N2)	218.124	0	20
L‐Glutamic acid (13C5;D5;15N)	159.103	20	40
L‐Asparagine (2,3,3‐D3)	136.078	40	20
L‐Valine (D5)	126.134	5	10
L‐Lysine (13C6)	153.129	10	20
L‐Glutamine (2,3,3,4,4‐D5)	152.110	20	0
L‐Glutamine (13C2)	149.081	10	20
Creatinine (N‐methyl‐D3)	117.088	10	20

### 2.2 Plasma sample preparation

Pooled human plasma, anti‐coagulated with citrate, was obtained from Sanquin Blood Bank (Leiden, the Netherlands). For protein precipitation, methanol was added to pooled human plasma at a 5:1 ratio. The plasma/methanol mixture was vortexed for 1 min at room temperature before centrifugation at 16100 g at 4°C for 10 min. Subsequently, 120 µL of the supernatant was transferred to an Eppendorf tube for liquid‐liquid extraction, for which 300 µL methanol, 450 µL chloroform, 140 µL water, 50 µL internal standard solution (200 µmol/L for L‐methionine and 60 µ µmol/L for DL‐phenyl‐D5‐alanine), and 50 µL isotope‐labeled compounds mix for classes I and II (50 µL water was used for the QC samples) were used to extract polar metabolites. Tables 1 and 2 provide an overview of how the samples were prepared for each class of plasma samples within study I and II, respectively. The samples were vortexed for 2 min and then centrifuged at 16100 g at 4°C for 10 min. 500 µL of the supernatant was centrifugally filtered using a 5 kDa cutoff filter (Millipore) at 12000 g at 4°C for 1.5 h to further remove proteins. The filtered sample was evaporated in a CentriVap Concentrator (Labconco) and stored at −80°C. The dried extract was reconstituted in 50 µL water prior to CE–MS analysis. Standards for calibration curves were generated by spiking the pooled human plasma with the mix of isotope labeled compounds at 10, 20, 40, 60, 80, and 100 µM, respectively.

### 2.3 CE–MS analysis

All fused‐silica capillaries used were 70 cm in length with an internal diameter of 50 µm and obtained from BGB Analytik (Harderwijk, the Netherlands). Prior to first use a newly installed capillary was conditioned using the following rinsing steps: water for 2 min at 5 bar, 0.1 M sodium hydroxide for 10 min at 5 bar, water for 2 min at 5 bar, and BGE for 2 min at 5 bar. The samples were injected hydrodynamically at 50 mbar for 20 s, which corresponds to circa 1.2% (∼17 nL) of the total capillary volume.

The analyses were conducted on an Agilent 7100 CE instrument hyphenated to an Agilent 6230 Time of Flight mass spectrometer (Agilent Technologies, Santa Clara, California), equipped with an ESI source via a co‐axial sheath‐liquid interface. The CE–MS approach used in this work was based on the work from Drouin et al.^23^ The sheath‐liquid, consisting of isopropanol/water (1:1, v/v) and acetic acid (200 µL added to a final volume of 100 mL sheath liquid), was delivered at a final flow‐rate of 5 µL/min by an Agilent 1260 Infinity II Isocratic Pump (Agilent Technologies) using a 1:100 splitter. A voltage of 30 kV was used for electrophoretic separation and detection was performed in positive MS mode. The MS parameters were as follows: drying gas was set at 100°C with a flow‐rate of 11 L/min, and the nebulizer gas at 0 psi. The capillary voltage was 5500 V, and the fragmentor, skimmer, and OCT1 RF voltages were set at 100, 50, and 150 V, respectively. The full scan MS acquisition covered the mass range from 50 to 1000 *m/z* at an acquisition rate of 1.5 spectra/s, which was controlled and monitored with MassHunter version B05.01 (Agilent). Between consecutive biological sample analyses, the capillary was flushed as follows: water for 30 s at 5 bar, methanol for 1 min at 5 bar, water for 30 s at 5 bar, 10% ammonium hydroxide for 1 min at 5 bar, water for 30 s at 5 bar and BGE for 2 min at 5 bar. The CE–MS data were stored as .d files.

The capillary cassette was thermostated at 22°C and the sample tray maintained at 10°C by means of a Julabo F12 circulator temperature controller (Boven‐Leeuwen, the Netherlands). To assess the repeatability of CE–MS for metabolic profiling of plasma, the RSD for migration time and peak area were determined for 19 endogenous metabolites in a QC sample, which was analyzed in 16 consecutive runs. During the analysis of the individual plasma samples, every ten runs a QC sample was analyzed. In total, 23 QC samples were analyzed in each study.

### 2.4 Data processing and chemometric analysis

An overview of the data analysis, by the software tools used in this study, is shown in Fig. 1. Each data analysis strategy is described in detail below.

#### 2.4.1 Strategy 1

The raw data were converted into mzXML format using ProteoWizard and imported into MZmine 2.32 for feature detection. The detailed detection process is listed in Supporting Information File S1. Considering that the peak area calculation function was not ideal in MZmine, the peak areas were calculated in the Data Acquisition module within MassHunter version B05.01 (Agilent). The peak areas were integrated based on a standard list generated by an untargeted analysis. Peak areas of the detected metabolites were corrected with the corresponding IS peak area (for study I with DL‐phenyl‐D5‐alanine and for study II with L‐methionine sulfone), and the peak area ratios were further used in the statistical analysis.

MetaboAnalyst (http://www.metaboanalyst.ca) was used for multivariate analysis, including principal component analysis (PCA) and partial least squares ‐ discriminant analysis (PLS‐DA) to identify the spiked markers as “biomarkers” to distinguish “class I” from “class II”. Auto‐scaling was done prior to PCA to prevent highly responsive metabolites from dominating the model, and prior to PLS‐DA to facilitate the discovery of the “spiked biomarkers”.^27^ The peak area ratios were also subjected to an unpaired non‐parametric test (Wilcoxon rank‐sum test, also known as Mann‐Whitney U test) within MetaboAnalyst, and false discovery rates (FDR) were calculated to discover if those *m/z* values are significant different between class I and II. The compounds responsible for distinguishing class I from class II samples were selected using the variable importance in projection (VIP) score employing the criteria of VIP > 1 and FDR < 0.05.

#### 2.4.2 Strategy 2

As in strategy 1, data was generated in centroid mode at an Agilent CE‐TOF‐MS instrument and converted to mzXML files with the open‐source file translator ProteoWizard. Compared to strategy 1, these files were imported and further analyzed in Matlab^TM^ R2014a (The Mathworks, Natick, MA) instead of MetaboAnalyst. Due to storage requirements, a binning method was necessary to compress the data^28^, ^29^ (Fig. 1). The regions‐of‐interest (ROI) method was used to compress the generated Total Ion Current profile.^30^ Here, ROI values are searched among all measurement times in the recorded CE–MS profile. However, different input variables are needed to define an ROI, such as a signal threshold value, mass accuracy and the minimum time interval to be considered as a peak width.^30^, ^31^ In our study, these parameters were set at 1000 for the signal threshold, mass accuracy was set to 0.01 Da and the minimum time to elute a peak was set to 6 s. All parameter values were based on the protocol by Gorrochategui et al.^30^ The following step was the feature detection step, which does not make use of MZmine, but is based on Multivariate Curve Resolution ‐ Alternating Least Squares (MCR‐ALS) using the MCR‐ALS toolbox. 32

As in strategy 1, peak areas were further integrated in the Data Acquisition module within MassHunter version B05.01 (Agilent) and corrected with the corresponding IS peak area (for study I with DL‐phenyl‐D5‐alanine and for study II with L‐methionine sulfone). The peak area ratios were further utilized in Matlab^TM^ R2014a (The Mathworks) to perform unsupervised PCA analysis, and supervised PLS‐DA analysis. Autoscaling was also applied here as data pre‐treatment method. The number of latent variables for the PLS‐DA model was chosen based on a five‐fold venetian‐blind cross validation. Additionally, the PLS‐DA model evaluation was based on the error rate, non‐error rate and accuracy, based on the cross‐validation and calibration results. Finally, compounds mainly responsible for distinguishing class I from class II samples were selected based on the VIP score, with the aim to hopefully trace back the spiked markers and confirm the results of strategy 1. An additional confirmation was performed with the same non‐parametric test as in strategy 1. All the *m/z* values resulting in a VIP value above 1 were analyzed with this univariate data analysis. Those resulting in a p‐value below 0.05 are significantly different between both classes and are important for distinguishing class I from class II samples.

## 3 Results and discussion

### 3.1 CE–MS for cationic metabolic profiling

Up till now, most metabolomics studies using CE–MS employed a standard co‐axial sheath‐liquid interface and low‐pH separation conditions to target cationic metabolites (i.e., basic compounds). In this study, this CE–MS approach was used in order to assess its capability of delivering proper chemical information in comparative metabolic profiling studies.

For comparative metabolic profiling, the CE–MS method should provide consistent migration times and peak areas over time. Therefore, pretreated blank pooled human plasma was first analyzed for 16 consecutive runs (lasting around 8 h in total). The RSD values for migration time, peak area, and peak area divided by IS, of 19 selected endogenous metabolites in this QC sample, were determined and are shown in Table 3. RSD values found are below 5.9, 9.1, and 4.5%, respectively. However, the lower RSD values are found for the corrected areas by the IS. For 16 of the 19 selected endogenous metabolites, the RSD values for migration time were below 3%. Therefore, we considered the overall findings acceptable to perform the proposed assessment study.

**Table 3:** Migration‐time and peak‐area repeatability (*n* = 16) for selected endogenous metabolites in pooled human plasma obtained by CE–MS. Abbreviations: MT, migration time

Compound	*m/z* value	MT RSD(%)	Area RSD(%)	Area ratio 1 RSD(%)*	Area ratio 2 RSD(%)*
Glycine	76.039	1.6	8.9	3.4	3.3
Serine	106.050	2.1	8.4	3.1	2.8
Proline	116.071	2.4	6.7	2.9	2.0
Valine	118.086	2.1	6.6	2.4	1.6
Threonine	120.066	2.3	7.9	3.3	3.1
Creatine	132.077	1.7	7.1	2.9	2.5
Asparagine	133.061	2.3	7.1	2.5	2.1
Ornithine	133.097	1.2	7.7	2.8	2.5
Glutamine	147.076	2.3	7.6	2.6	2.2
Glutamic acid	148.060	2.4	6.9	2.9	2.4
Phenyl‐D5‐alanine (IS2)	171.123	2.4	6.1	NA	NA
Arginine	175.119	1.3	7.2	3.5	3.0
L‐Methionine Sulfone (IS1)	182.048	2.7	6.6	NA	NA
L‐Alanine	90.055	1.8	8.2	3.6	3.0
L‐Isoleucine	132.102	4.4	4.4	4.2	3.0
L‐Leucine	132.102	5.9	5.9	2.3	1.1
L‐Lysine	147.113	1.2	7.4	2.6	2.0
L‐Methionine	150.058	2.3	9.1	4.3	4.5
L‐Histidine	156.077	1.4	6.5	2.4	2.4
L‐Phenylalanine	166.086	2.4	6.1	2.8	1.5
L‐Tyrosine	182.081	2.5	6.9	4.0	3.3

^*^Area ratio 1 is representing the corrected areas for the first internal standard, L‐Methionine Sulfone. The second internal standard is Phenyl‐D5‐alanine and the correction for this internal standard resulted in the RSD values of area ratio 2.

### 3.2 Suitability of CE–MS for metabolic profiling of human plasma

To assess whether the CE–MS method for cationic metabolic profiling has the capability to deliver proper chemical information in biomarker discovery studies, a metabolomics study was simulated. For this purpose, isotope‐labeled compounds were used as “spiked biomarkers”, while the capability of CE–MS to trace them back as biomarkers was examined. The selected isotope‐labeled compounds included diverse chemical structures and were evenly spread over the analysis time. Another requirement was that the unlabeled form could be observed with a good detection sensitivity by CE–MS. Prior to performing the simulation study, some performance metrics of CE–MS for the analysis of the selected isotope‐labeled compounds were determined. Special focus was on the accuracy of the method. The accuracy was determined comparing the spiked concentrations of the isotope‐labeled compounds, with those experimentally estimated using calibration curves. The accuracy for all labeled compounds was found to be in the range of 85% to 115% (Supporting Information Table S1).

Study I (Table 1) focused on analyzing three sets of plasma samples, i.e., class I is spiked with five isotope‐labeled compounds, class II is spiked with six different isotope‐labeled compounds, and set three consists of blank pooled human plasma (used as QC). In order to mimic a comparative metabolomics study, samples were constructed in a way as indicated in Table 1, in which the (introduced) concentration differences for the spiked compounds between the plasma samples can be found. In metabolomics, it is important to include QC samples to provide information about the robustness of the method^33^ and to mimic the sample composition, qualitatively and quantitatively.^34^ Study II (Table 2) focused on more subtle differences by spiking the ‘markers’ in both groups with concentrations which differ by a factor 2 between both classes (Table 2), and with the absence of one compound in each class. For comparative metabolic profiling only compounds with RSD values for migration time and corrected peak area below 5 and 30%, respectively, as calculated for each class including QC samples (*n* = 23), were considered for data analysis as those with higher values may be considered as spurious signals.^35^ Supporting Information Fig. S1 shows extracted ion electropherograms obtained by CE–MS for the analysis of the spiked compounds in plasma samples of Group 2, Study II. Supporting Information Fig. S2 shows extracted ion electropherograms obtained for the analysis of selected endogenous compounds in a QC sample by CE–MS (Supporting Information Fig. S2A) including a mass spectrum for the same time window after noise subtraction (Supporting Information Fig. S2B).

#### 3.2.1 Data analysis for study I

The design of this first study introduced two groups of metabolites into individual classes, so it was merely the absence/presence of differences that needed to be distinguished. The whole corrected data matrix for the IS, including all the samples, which differs in composition of the mixtures mentioned in Table 1, are used for further data analysis.

The first feature detection approach with MZmine (from Strategy 1) resulted in more than 100 features. A feature is defined as a given mass‐to‐charge number with a defined migration time and intensity. Manual examination was then introduced to exclude falsely identified features, resulting in 70 features with peak heights above 1000. Except for the 11 compounds used for spiking, all the corrected peak areas detected in QC samples and class I and II samples showed variation far below 30% in RSD.

Strategy 2 does not need alignment of the peaks and is therefore suitable for CE data where, especially the late‐migrating analytes, may experience significant migration shifts between samples.^31^ 67 features were investigated, resulting in the parameters for the best MCR‐ALS model, with an explained variance of 99.1% and an lack‐of‐fit value of 9.3%. For 67 resolved compounds, which can be related to endogenous metabolites or spurious markers, the RSD values for corrected peak areas and migration times were maximally 29.0 and 3.8%, respectively.

**Figure 2:**
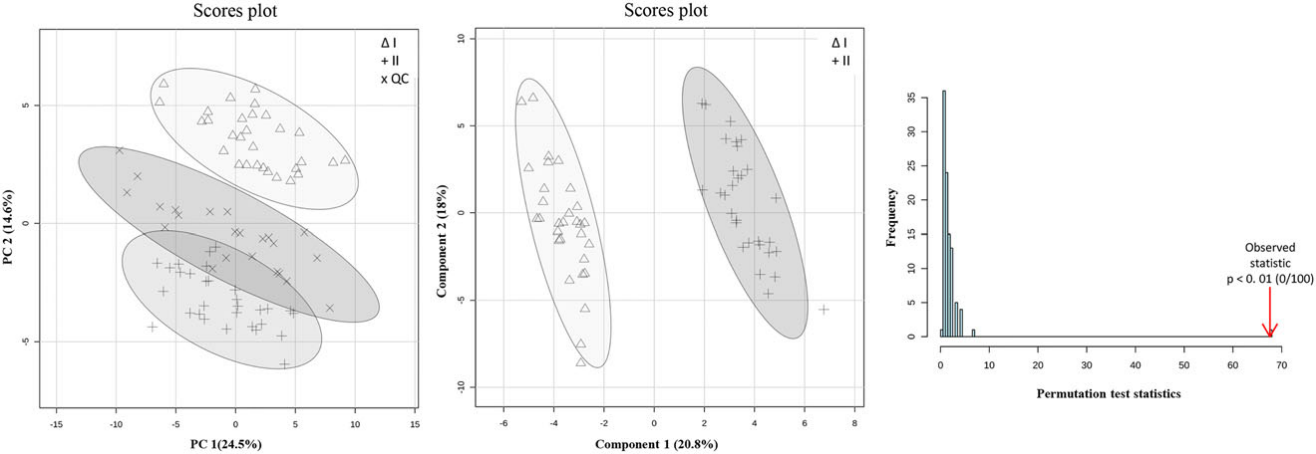
Multivariate results for study I obtained with MetaboAnalyst 4.0. (A) PC1‐PC2 score plot for the area corrected by the IS. △, + and ✕ symbols represent samples of class I, class II and QC group, respectively. The elliptic areas represent the 95% confidence regions; (B) PLS‐DA scores plot. △ and + symbols represent samples of class I and II, separately; (C) Permutation test results of the PLS‐DA model (statistical test: separation distance (B/W)), number of permutations set at 100.

**Figure 3:**
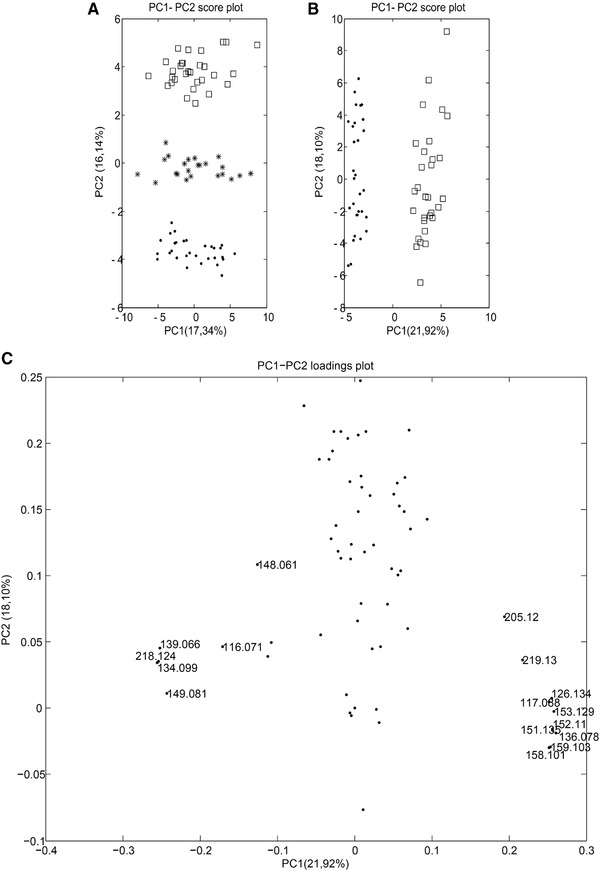
A) PC1‐PC2 score plot obtained for the **X** matrix of study I of the second data analysis strategy using internal standard correction and autoscaling; Quality Control samples are represented by stars; Class I by dots and Class II by squares; (B) PC1‐PC2 score plot for the two groups using internal standard correction and autoscaling; (C) PC1‐PC2 loadings plot (for numbers see Supporting Information Table S2).

PCA was first conducted to investigate relations between groups. Auto‐scaling was adopted as data‐pretreatment to strip away the dominance of highly responsive/abundant metabolites and to render all metabolites equally important. PCA plots thus generated from study I, using both data‐analysis approaches, are displayed in Figs. 2A and 3A. Good separation of the three groups was observed in both cases. However, Fig. 3A will result in better separation of the groups, which may be the result of a different number of features in the **X**‐matrix resolved by another feature selection method. It is worth noticing that samples in all groups in both PCA plots sprawled mainly along PC1, suggesting that most variation could be explained by the instrumental drift, while the difference between the groups was along PC2. However, no QC correction was performed because of the lack of spiked markers in the QC sample, which are pooled human plasma samples. The two spiked groups were well separated in Fig. 3B. Then a supervised analysis is performed to build a classification model and to identify the features responsible for the classification.

PLS‐DA is a commonly used classification method in metabolomics studies, because of its ability to identify biomarkers from the loadings of the model.^29^ In the first data analysis strategy with MetaboAnalyst, a five‐component PLS‐DA model was established based on the leave‐one‐out cross validation (LOOCV) results. The obtained PLS‐DA plot is shown in Fig. 2B. The LOOCV parameters, R^2^ = 0.994 and Q^2^ = 0.979, indicated an excellently fitting and predictive PLS‐DA model. In order to prevent PLS‐DA from overfitting the data, the established model was validated by performing a permutation test to determine whether differences observed between groups are significant.^36^, ^37^ In each permutation, a PLS‐DA model is established between the data (**X**) and the permuted class labels (**y**), utilizing the previously determined optimal number of components. Then the ratio of the between‐group sum of the squares and the within‐group sum of squares, indicated as B/W‐ratio, is calculated for the class assignment predictions of each PLS‐DA model built. These ratios can be plotted in a histogram known as “the distribution of random class assignments”.^36^ If the B/W ratio of the original class assignment is part of this distribution, the differences between the two class assignments cannot be deemed significant. In the permutation test in strategy 1, the class assignment was permuted 100 times (histogram shown in Fig. 2C). The bar pointed out by the arrow represents the original sample. A p‐value below 0.01 in 100 permutations means that not even once (<0.01*100) did the permutated data yield better performance (higher B/W) than the original label, suggesting the significant difference between these two classes.

The second data analysis approach resulted in a less complex PLS‐DA model with only one latent variable, based on the values for the non‐error rate and the not‐assigned samples. The PLS‐DA model was evaluated by five‐fold venetian blind cross‐validation, instead of LOOCV, because the latter may over‐estimate the predictive power. Good merits of the model were demonstrated with an excellent predictive ability of 100% accuracy and a zero‐error rate. Comparing the two PLS‐DA models shows a simpler model with Strategy 2, which is the result of the better separation of the two classes observed in the unsupervised PCA plot in Fig 3A.

The validation of supervised models is often lacking in metabolomics studies.^28^, ^29^, ^38^, ^39^ The validation of the established models in both strategies was performed with cross‐validation, LOOCV or five‐fold venetian blind cross‐validation, respectively. These cross‐validation approaches are often conducted when only a limited number of samples are involved, as in the present study, but it was also reported that this approach may have the risk of over‐fitting, especially LOOCV.^29^

VIP scores are often applied to select variables that are important in the projection in PLS‐DA models and for the differentiation of the groups. A variable with a VIP value above 1.0 may be considered important.^40^ Data analysis strategy 1 revealed 17 features and strategy 2 revealed 16 features (Supporting Information Table S2) with VIP scores above 1.0. In the results from both data analysis strategies, the 11 spiked “markers” were detected with VIP scores above 2.0. The FDR were obtained from the unpaired non‐parametric test in order to assess the incidence of false positives. All 17 features had FDR values below 0.05 (data not shown), indicating that these features can indeed be regarded as potential “markers”.

**Figure 4:**
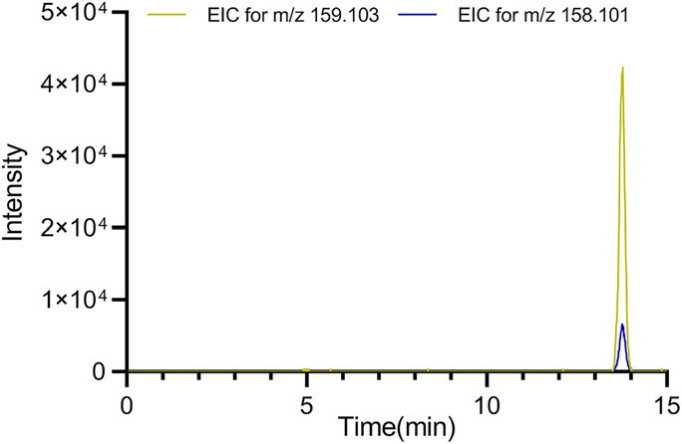
Extracted ion electropherogram obtained by injecting the standard solution of compound *m/z* 159.103, resolving the contaminant *m/z* 158.101.

The second data analysis strategy took also into consideration the results generated from the non‐parametric test to confirm whether the results of the VIP score for the defined features were significantly different for comparing class I with class II, and it resulted in p‐values below 0.0001 for all 16 *m/z* values. Furthermore, the PC1‐PC2 loadings plot (Fig. 3C) showed similar findings as the statistical tests, i.e., five extra features (9, 13, 14, 20, 21), apart from the 11 spiked compounds are among the highest absolute loadings, indicating their contribution to the group classification. Among these detected features, *m/z* 158.101 showed a comparable VIP score to the rest of the spiked features in data analysis strategy 2. The individual standard solutions of the spiked compounds were injected and analyzed in an attempt to determine the source of feature *m/z* 158.101. Fig. 4 clearly shows that *m/z* 158.101 and *m/z* 159.103 are detected at the same migration time, thereby suggesting that *m/z* 158.101 could potentially be another labeled form of the same original compound (L‐Glutamic acid). The reason why this feature was not detected in strategy 1 is that the peak height of *m/z* 158.101 did not always meet the peak height threshold of 1000, and got omitted from the feature list by the filtering function within MZmine.

Apart from the features discussed above, there are still some unaccounted features with a VIP score above 1.0. However, the reason why these variables ended up being “markers” is not clear at this stage. Strategy 2 resulted in 5 unaccounted markers (9, 13, 14, 20, 21), which could be related to an impurity. Strategy 1 resulted in 6 spurious markers (13, 15–19). Strategy 2 resulted in better results for all steps performed in study I. The separation of the different groups was clearer, the PLS‐DA model was much simpler for a better performance and less unknown markers are indicated. In the future, it will be interesting to investigate the importance of the unaccounted markers in more detail.

#### 3.2.2 Data analysis for study II

Study I showed that spiked “markers” were detected by both data analysis strategies, but it is important to stress that in real‐life metabolomics studies, changes in the abundance of metabolites tend to be more subtle than those introduced in study 1, where spiked metabolites were present in one group and not in the other. In the second study more subtle differences (Table 2) were introduced between the two classes, which anyway still might be larger than the very small metabolic differences that may actually occur between healthy and diseased individuals.

The data from the second study were subjected to the same analysis processes as study I. The application of MZmine resulted in 73 features, among which only 3 features had RSD values above 30%. Those features were deleted prior to further data analysis. The MCR‐ALS model in strategy 2 resulted in 90 features with 99.2% explained variance and 9.2% lack‐of‐fit. After removing features with RSD values of peak area ratios over 30%, 84 remained in the data set.

**Figure 5:**
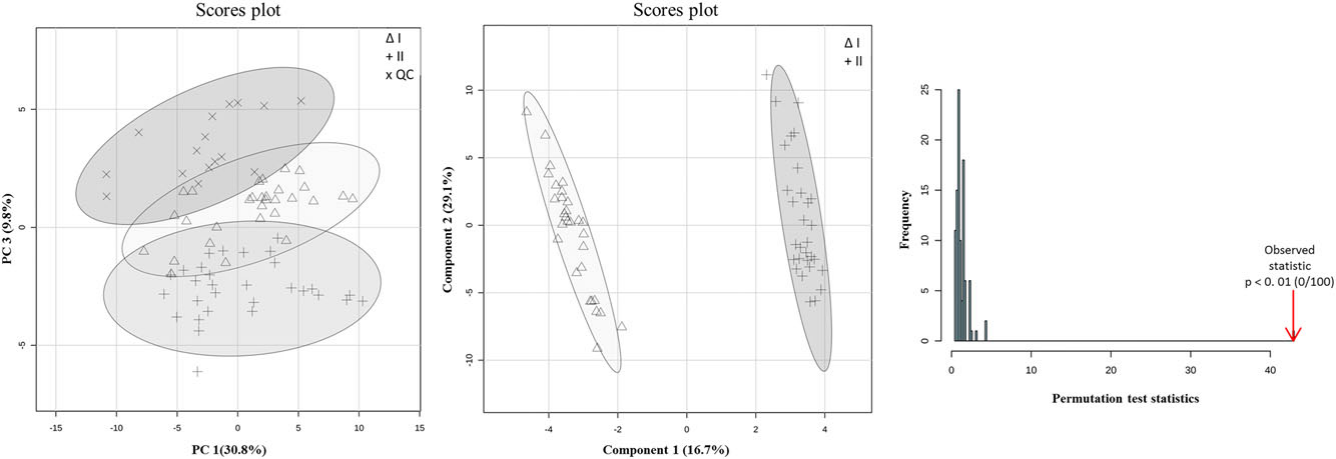
Multivariate results for study II obtained with MetaboAnalyst 4.0. A) PC1‐PC3 score plot for the area corrected by the IS. △, + and ✕ symbols represent samples of class I, class II and QC group, respectively. The elliptic areas represent the 95% confidence regions; B) PLS‐DA scores plot. △ and + symbols represent samples of class I and II, respectively; C) Permutation test results of the PLS‐DA model (statistical test: separation distance (B/W)), number of permutations set at 100.

**Figure 6:**
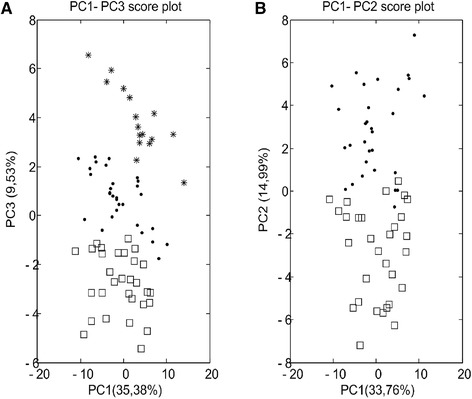
A) PC1‐PC3 Score plot of study II obtained with the second data analysis strategy using internal standard correction and autoscaling. Quality Control samples are represented by stars, class I by dots and class II by squares; B) PC1‐PC2 Score plot for the two groups using internal standard correction and autoscaling.

PCA score plots were generated after auto‐scaling the peak area ratios in both strategies, as shown in Figs. 5A and 6A. As in study I, the QC samples were distributed along PC1, indicating that the largest variation in the first PC was not related to the group information. The auto‐scaled data were well separated along PC3. The PC1‐PC2 score plot for only the two spiked groups (Fig. 6B) shows that these groups tend to be separated, despite the subtle differences between the profiles.

A PLS‐DA model with five components was established in the first data analysis strategy, using MetaboAnalyst, with parameters R^2^ = 0.998 and Q^2^ = 0.995 acquired by LOOCV. A 100‐ permutations test using separation distance (B/W) as test parameter, was performed with the observed statistic having a p<0.01 (Fig. 5C). The second data analysis strategy resulted in a PLS‐DA model with one latent variable, with good predictive ability (100% accuracy and non‐error rate) and the model was validated as mentioned above by the same cross‐validation method, resulting in good parameters (100% accuracy and non‐error rate). Based on the criteria VIP>1.0, and FDR or p<0.05 in the non‐parametric tests in both data analysis strategies, the 11 spiked compounds were identified as “biomarkers” in study II (Supporting Information Table S3). However, again in Strategy 2 the peak with *m/z* value 158.101 was indicated as a biomarker.

Again features with peak heights over 1000 were extracted for further data analysis, because smaller peaks are difficult to measure precisely and might increase the chance of false biomarker identification.^41^, ^42^ For a reliable detection of low abundant metabolites with the current CE–MS set‐up, the use of an in‐capillary preconcentration technique is needed.^43^, ^44^

In summary, both data processing and analysis strategies resulted in similar findings, despite the small differences observed with the VIP scores. An interesting phenomenon is that the three groups were better separated in the PCA score plots using the second strategy. Additionally, the better separation may be the result of simpler PLS‐DA models in the second strategy compared to the 5 component PLS‐DA model in the first strategy. This might be the consequence of the different numbers of *m/z* values included in the **X**‐matrix. However, the better separation results between the groups with data analysis strategy 2 makes this method more suitable. However, strategy 2 is more time consuming and more difficult for an analyst less skilled with data analysis approaches. In real‐life cases, targeted metabolomics studies are usually required after untargeted analysis, quantifying the earlier indicated potential “biomarkers”, in order to validate and quantify the extent of changes in “biomarkers”. The essential message here is that all spiked features could be distinguished by univariate and multivariate analyses of the recorded sheath‐liquid CE–MS data. This clearly emphasizes the utility of sheath‐liquid CE–MS in metabolomics studies of human plasma samples.

## 4 Conclusions and perspectives

In metabolomics, CE–MS has become a useful analytical technique for the profiling of highly polar and charged compounds. In the context of biomarker discovery, it is important to assess whether a given analytical technique provides the proper chemical information and does not result in false positive or negative decisions. In this study, the utility of CE–MS for this purpose was evaluated. Different chemometric analysis procedures were used in order to confirm each other's results and to show that both data analysis strategies give similar information. As shown, the second strategy will indicate less spurious markers in study I and shows a better separation between the groups in study II. However, the latter approach is more difficult to perform than the use of the MetaboAnalyst software.

Additionally, in this work the two data analysis strategies resulted in very similar outcomes, as expected, and showed that CE–MS in combination with data analysis tools may help to uncover the spiked “biomarkers”. Overall, this work emphasized the capability of CE–MS in metabolic profiling studies of human plasma. The usefulness of CE–MS for comparative metabolic profiling may also be evaluated using a comparison or cross‐validation with another analytical technique, such as, for example HILIC‐MS or NMR spectroscopy. In this case it would be important to focus in such a study on the compounds that can be covered by each analytical technique. For a follow‐up study, it would also be interesting to use very small differences in concentration levels for the spiked compounds between sample groups in order to better simulate the actual biological situation in which metabolic differences may be very subtle or to make use of real‐life samples.

*The authors have declared no conflict of interest*.

## Supporting information

Supporting Information

Supporting Information

Supporting Information

Supporting Information

Supporting Information
