# Supplementary material for: Assessing the suitability of capillary electrophoresis‐mass spectrometry for biomarker discovery in plasma‐based metabolomics
Source: Electrophoresis. 2019 May 2;40(18-19):2309–20. doi: 10.1002/elps.201900126 (PMC6767474; doi:10.1002/elps.201900126)
Supplement: Supplementary file 1 — Supporting Information [file ELPS-40-2309-s001.docx]

**Assessing the actual utility of capillary electrophoresis-mass spectrometry for metabolic profiling of human plasma**

Wei Zhang^1#^, Karen Segers^1-3#^, Debby Mangelings^2^, Ann van Eeckhaut^3^, Thomas Hankemeier^1,4^, Yvan Vander Heyden^2^, Rawi Ramautar^1*^

MZMine 2.32 procedure and settings

Raw data import

Peak detection

- Mass detection:
- Mass detector: Centroid
- Noise level: 10^3
- MS level:1
- Chromatogram builder:
- Min time span(min): 0.02
- Min height: 10^3
- M/Z tolerance: 0.01 m/z or 12 ppm
- Retention time: 5-20 min
- Chromatogram deconvolution:
- Algorithm: Local minimum search
- Chromatographic threshold: 65%
- Search minimum in RT range (min): 0.05
- Minimum relative height: 5%
- Minimum absolute height: 1000
- Min ratio of peak top/edge: 2
- Peak duration range(min): 0.05-0.8

Alignment

- Retention time normalizer:
- MZ tolerance: 0.01 m/z or 12 ppm
- Retention time tolerance: 15% Relative
- Minimum standard intensity: 1000
- Alignment: RANSAC Aligner
- MZ tolerance: 0.01 m/z or 12 ppm
- RT tolerance: 10% Relative
- RT tolerance after correction: 5% Relative
- RANSAC iterations 10000
- Minimum number of points 20%
- Threshold value: 0.05
- Linear model: No
- Filtering: peak list rows filter
- Minimum peaks in a row: 14
- Minimum peaks in an isotope pattern: 0
- m/z: auto range
- Retention time: 5-20 min
- Peak duration range: 0-0.8
- Gap filling: Same RT and m/z gap filler
- MZ tolerance: 0.005 m/z or 12 ppm
- Filtering: duplicate peak filter
- MZ tolerance: 0.005 m/z or 12 ppm
- RT tolerance: 5%
