# Supplementary figures and images for: Assessing the suitability of capillary electrophoresis‐mass spectrometry for biomarker discovery in plasma‐based metabolomics
Source: Electrophoresis. 2019 May 2;40(18-19):2309–20. doi: 10.1002/elps.201900126 (PMC6767474; doi:10.1002/elps.201900126)

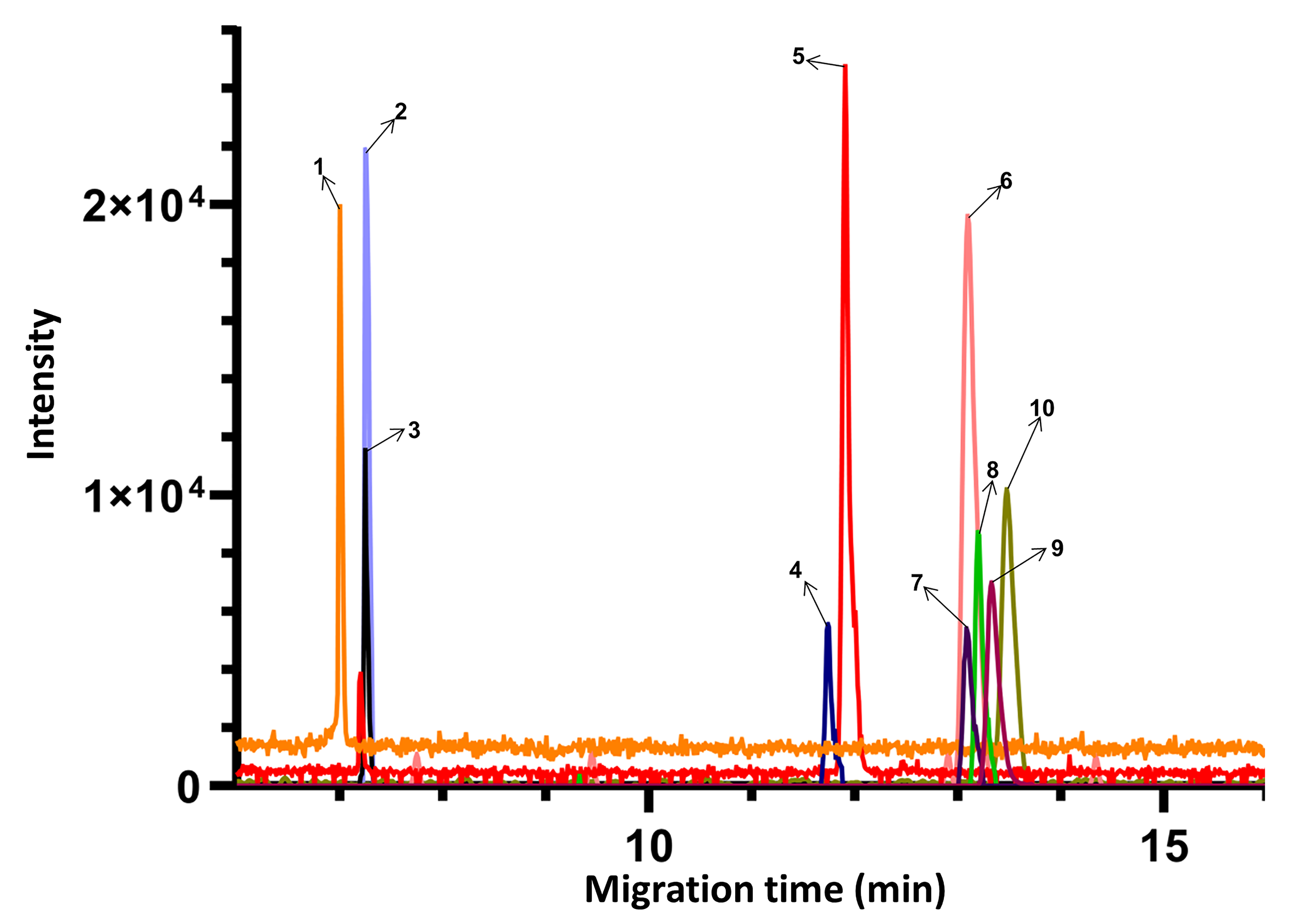

Supplement: Supplementary file 2 — Supporting Information [file ELPS-40-2309-s002.tif]

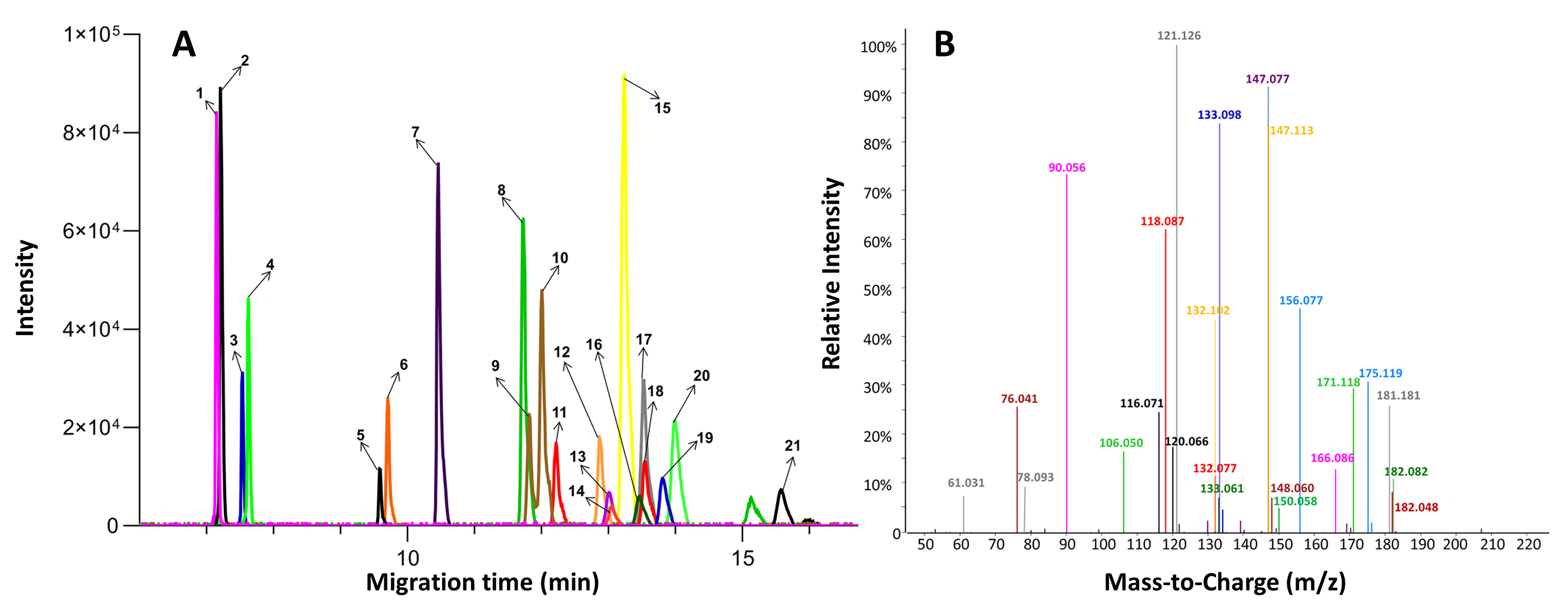

Supplement: Supplementary file 3 — Supporting Information [file ELPS-40-2309-s003.tif]
