# Supplementary material for: Assessing the suitability of capillary electrophoresis‐mass spectrometry for biomarker discovery in plasma‐based metabolomics
Source: Electrophoresis. 2019 May 2;40(18-19):2309–20. doi: 10.1002/elps.201900126 (PMC6767474; doi:10.1002/elps.201900126)
Supplement: Supplementary file 4 — Supporting Information [file ELPS-40-2309-s004.docx]

| Metric | Creatinine(N-methyl-D3) | L-Asparagine (13C2;15N2) | L-Asparagine (2,3,3-D3) | L-Glutamic acid (13C5;D5;15N) | L-Glutamine (13C2) | L-Glutamine (2,3,3,4,4-D5) | L-Isoleucine (13C; 15N) | L-Lysine (13C6) | L-Lysine(4,4,5,5-D4) | L-Tryptophan (13C11;15N2) | L-Valine (D5) |
| --- | --- | --- | --- | --- | --- | --- | --- | --- | --- | --- | --- |
| Determination coefficient*  (R^2^) | 0.9992 | 0.9909 | 0.9973 | 0.9995 | 0.9987 | 0.998 | 0.9992 | 0.9991 | 0.9991 | 0.9988 | 0.9991 |
| Precision (%RSD) (n=3) | 8.1% | 3.0% | 7.5% | 4.2% | 5.3% | 5.1% | 7.7% | 9.5% | 12.8% | 4.8% | 3.7% |
| Accuracy* (n=3) | 106.1% | 89.4% | 98.9% | 103.9% | 95.9% | 98.9% | 108.7% | 98.9% | 100.7% | 104.9% | 102.2% |
|  | 99.4% | 90.7% | 92.3% | 104.3% | 93.2% | 97.1% | 107.0% | 91.5% | 87.2% | 104.6% | 102.9% |
|  | 95.3% | 88.0% | 95.6% | 99.0% | 93.2% | 99.2% | 97.1% | 88.4% | 86.4% | 98.9% | 99.3% |

**Supplementary Table S1.** CE-MS performance metrics for the analysis of isotope-labeled compounds in plasma. Precision of peak areas was based on the analysis of three plasma samples spiked with 40 μM of the labeled compounds.

*Peak areas were corrected with IS2. Linearity determined in the range from 10 to 100 μM.

**Supplementary Table S2.** Variable Importance in Projection (VIP) scores obtained for study I using data analysis strategies 1 and 2. The reported m/z values have VIP values above 1.0.

| Number | *m/z* value | Compound | VIP score strategy 1 | VIP score strategy 2 |
| --- | --- | --- | --- | --- |
| 1 | 152.110* | L-Glutamine (2,3,3,4,4-D5) | 2.27 | 2.17 |
| 2 | 151.135* | L-Lysine (4,4,5,5-D4) | 2.16 | 2.17 |
| 3 | 136.078* | L-Asparagine (2,3,3-D3) | 2.26 | 2.17 |
| 4 | 218.124* | L-Tryptophan (13C11;15N2) | 2.25 | 2.15 |
| 5 | 134.099* | L-Isoleucine (13C;15N) | 2.24 | 2.14 |
| 6 | 153.129* | L-Lysine (13C6) | 2.23 | 2.14 |
| 7 | 139.066* | L-Asparagine (13C2;15N2) | 2.14 | 2.12 |
| 8 | 159.103* | L-Glutamic Acid (13C5;D5;15N) | 2.20 | 2.11 |
| 9 | 158.101 | Unknown | NA | 2.09 |
| 10 | 126.134* | L-Valine (D5) | 2.14 | 2.09 |
| 11 | 117.088* | Creatinine (N-methyl-D3) | 2.18 | 2.08 |
| 12 | 149.081* | L-Glutamine (13C2) | 2.13 | 2.05 |
| 13 | 116.071 | Proline | 1.33 | 1.27 |
| 14 | 148.061 | Glutamic acid | NA | 1.16 |
| 15 | 158.154 | Unknown | 1.49 | NA |
| 16 | 148.079 | Unknown | 1.30 | NA |
| 17 | 246.205 | Unknown | 1.06 | NA |
| 18 | 184.170 | Unknown | 1.24 | NA |
| 19 | 104.111 | Unknown | 1.30 | NA |
| 20 | 219.130 | Unknown | NA | 1.58 |
| 21 | 205.120 | Unknown | NA | 1.37 |

* These *m/z* values are related to the 11 spiked compounds. NA, not applicable

**Supplementary Table S3.** Variable Importance in Projection (VIP) scores obtained for study II using data analysis strategies 1 and 2. All *m/z* values have VIP values above 1.0.

| *m/z* value | Compound | VIP score strategy 1 | VIP score strategy 2 |
| --- | --- | --- | --- |
| 218.124* | L-Tryptophan (13C11,15N2) | 2.50 | 2.63 |
| 136.078* | L-Asparagine (2,3,3-D3) | 2.47 | 2.60 |
| 159.103* | L-Glutamic acid (13C5;D5;15N) | 2.38 | 2.57 |
| 126.134* | L-Valine (D5) | 2.45 | 2.57 |
| 149.081* | L-Glutamine (13C2) | 2.45 | 2.57 |
| 158.101 | Unknown | NA | 2.57 |
| 134.099* | L-Isoleucine (13C;15N) | 2.46 | 2.55 |
| 139.066* | L-Asparagine (13C2;15N2) | 2.46 | 2.55 |
| 151.135* | L-Lysine (4,4,5,5-D4) | 2.41 | 2.53 |
| 117.088* | Creatinine (N-methyl-D3) | 2.35 | 2.50 |
| 153.129* | L-Lysine (13C6) | 2.38 | 2.47 |
| 152.110* | L-Glutamine (2,3,3,4,4-D5) | 2.49 | 2.44 |

* These *m/z* values are related to the 11 spiked compounds. NA, not applicable.
