# Supplementary material for: Assessing the suitability of capillary electrophoresis‐mass spectrometry for biomarker discovery in plasma‐based metabolomics
Source: Electrophoresis. 2019 May 2;40(18-19):2309–20. doi: 10.1002/elps.201900126 (PMC6767474; doi:10.1002/elps.201900126)
Supplement: Supplementary file 5 — Supporting Information [file ELPS-40-2309-s005.docx]

Supplementary Figure 1. Extracted ion electropherograms obtained by CE-MS for the analysis of spiked compounds in plasma samples from Group 2, Study II. The highlighted compounds are: 1. Creatinine (N-methyl-D3); 2. L-Lysine (13C6); 3. L-Lysine (4,4,5,5-D4); 4. L-Valine (D5); 5. L-Isoleucine (13C; 15N); 6. L-Asparagine (13C2;15N2); 7. L-Asparagine (2,3,3-D3); 8. L-Tryptophan (13C11;15N2); 9.L-Glutamine (13C2); 10. L-Glutamic acid (13C5;D5;15N).

Supplementary Figure 2. Extracted ion electropherograms (A) obtained for selected endogenous compounds in a QC sample from Study II by CE-MS, and mass spectrum (B) extracted from the same time window after noise subtraction. The highlighted compounds are as follows: 1. Ornithine (m/z 133.098); 2. L-Lysine (m/z 147.113); 3. Arginine (m/z 175.119); 4. L-Histidine (m/z 156.077); 5. Creatine (m/z 132.077); 6. Glycine (m/z 76.041); 7. L-Alanine (m/z 90.056); 8. Valine (m/z 118.087); 9. L-Isoleucine (m/z 132.102); 10. L-Leucine (m/z 132.102); 11. Serine (m/z 106.050); 12. Threonine (m/z 120.066); 13. Asparagine (m/z 133.061); 14. L-Methionine (m/z 150.058); 15. Glutamine (m/z 147.077); 16. Glutamic acid (m/z 148.060); 17. Phenyl-D5-alanine (IS2) (m/z 171.118); 18. L-Phenylalanine (m/z 166.086); 19. L-Tyrosine (m/z 182.082); 20. Proline (m/z 116.071); 21. L-Methionine sulfone (IS1) (m/z 182.048).
